# Surgery

**Published:** 1909-06

**Authors:** C. A. Morton


### SURGERY.

IN a report on 2,000 cases of operation for **Radical Cure of Hernia,** Bull and Coley, of New York,[1] have given us the results of the operations performed at the Hospital for the Crippled and Ruptured, from 1890 to 1907, by Drs. Bull, Coley and Walker, and the Assistant Surgeons. They were almost all on children, only 137 were operations on adults. The method of reporting the results in this paper is not wholly satisfactory. We are told that there were so many relapses out of a certain number of cases, but we are not told for how long the cases were followed to see if relapse occurred. Thus we are told that there were only nine failures to obtain a cure in 1,185 cases of Bassini's operation, but not whether an attempt was made to find out the late result in all these cases, and, if so, in how many cases the patient was traced, and at what period after operation he was found free from recurrence. But we are told that 837 cases of operation for inguinal hernia were traced for periods of from one to fourteen years, and the results are given. Therefore, evidently the whole of the 1,185 cases were not traced even for one year, and we may presume that the nine relapses in the 1,185 cases were simply relapses that happened to come under observation. This, of course, cannot represent the true proportion.

However, there is much useful information to be obtained from this paper, though the statistics are not all that we should desire. The authors consider that it makes very little difference in the result whether, in doing Bassini's operation, the cord is placed in a superficial relation to the internal oblique after it has been sutured to Poupart's ligament, or is left in its natural position. They operated without transplantation of the cord in 346 cases. The results of Bassini's operation in their hands has evidently been an encouraging one; but in children some surgeons consider that it is not necessary to do as much as this, and that simply a high ligature of the sac is all that is necessary. Mr. Harold Stiles[2] has reported 360 cases of operation for hernia in infants and young children, with only ligature of the neck of the sac at the external ring, and the use of a suture drawing the pillars of the ring and the conjoint tendon together; and he only knows of four relapses, and two of these were after radical

[1] *J. Am. M. Ass.*, 1907, xlix, 1017. [2] *Brit. M. J.*, 1904, ii, 812.

cure, and one after operation for strangulation. Stiles does not give us any series of statistics as to recurrence—he says many of his cases had been too recently operated on to do so—but neither do Bull and Coley give a consecutive series of traced cases to support their statistics.

As to the mortality of the operation in children, Bull and Coley's cases are most encouraging, for they only had one quarter of 1 per cent. mortality in 1,978 cases, and, as already stated, almost all these were operations on children. Stiles's mortality was also very small, five deaths in the 360 cases, and one of these was due to chloroform, another followed operation for strangulation; 26 per cent. were on infants under one year old. Drs. Bull and Coley used kangaroo tendon, chromicised to last three to four weeks, in almost all their cases. They operated on all their cases of femoral hernia with a purse string suture of kangaroo tendon applied to the crural canal, and it is stated that one of the authors has operated on 125 cases without a relapse, but no statistics are given as to the number of cases traced for any given period. They do not advise operation under four years, as they consider that the risk is greater than in children older than four, and they believe that a considerable number of children under four are cured by a truss, and that strangulation under that age is very rare. Stiles's statistics show that the risk is very small in operations in infancy, and that when strangulation does occur in childhood (which is rare) it nearly always occurs in infancy. He disagrees with Bull and Coley that in a considerable number of children the rupture can be cured by a truss. But statistics showing the result of truss treatment at different ages in infancy and childhood, for given periods, are much wanted. That it often fails there can, of course, be no doubt; but we want reliable data as to how often it succeeds. Even after the rupture seems cured the case must be watched for a considerable time before being reported, as a rupture may suddenly return a long time after the sac has been supposed to be obliterated by the pressure of a truss, when there is some extra strain or a severe cough.

**The Pathology and Treatment of Inguinal Hernia in Children** has been discussed by Mr. C. H. Fagge[1] in a recent paper based on his experience of seventy-eight cases at the Evelina Hospital. He accepts the view of Hamilton Russell, of Melbourne,[2] that the sac is always congenital and not acquired, with regard to these hernias in children, and that the hernia occurs into a non-obliterated funicular process. In only eight of his seventy-eight cases was the hernia what is generally called a congenital one, *i.e.* a hernia into the tunica vaginalis around the testis. He thinks it much more likely that bowel would descend into such an unobliterated funicular process, than that it would push out

[1] *Lancet*, 1908, ii, 1270. [2] *Lancet*, 1899, i, 1353; 1902, i, 1519.

a new hernial peritoneal sac, through a weak place in the abdominal wall in a young child, apparently because he does not think such a weak spot likely to exist. He does not believe that hernia, even in a baby, is ever translucent, for he considers such cases are really unrecognised hydroceles.

The most important matter with regard to hernia in children, which he considers at length, is the question of operation. He is much in favour of operating even in babies, in all cases. But he does not make it quite clear whether he has any hope of curing them with a truss, for he says: "I think it is now the duty of every medical man to impress upon parents that trusses hold out no prospect of permanent cure, as the time has certainly arrived when cure can be more easily and almost certainly obtained by operative means, which in themselves are practically devoid of risk." If the cure can be "more easily and certainly obtained" by operation, this implies a chance of cure by trusses, and therefore the surgeon could not say to any parent they "hold out no prospect of permanent cure." But he elsewhere in the paper says that he cannot believe trusses ever cure ruptures, though they may prevent their descent; and he states that "of all the patients whom, as an out-patient surgeon, I treated here,[1] I can only remember one in whom the treatment had been so consistently carried out that, after an interval of two years or more, the rupture no longer came down when the patient stood up without the truss." We should, of course, prefer some statistics on the matter, rather than the statement in this form, as many hospital cases are never followed for two years after truss treatment is undertaken. If the hernia no longer came down, it seems quite likely the parents would leave off the truss, and would no longer take the child to the hospital. He seems to consider that the pressure of a truss will never obliterate the sac, so that the rupture may at any time descend into it. But, as before pointed out, he is not quite consistent, for he says: "A truss must be worn day and night for several years before we can hope that the hernia will not return if the truss is omitted, and during this time great care must be taken that a new truss is obtained as soon as the child outgrows the old one. It is a matter of common experience in all hospital out-patient departments that the patients soon tire of the trouble and expense, and allow the hernia to go its own way." This certainly seems to imply a belief that if truss treatment is efficiently carried out for a long enough period it may cure the hernia, for if the rupture never returns that is a practical cure.

Mr. Fagge is in favour of operating after the first few months of life on all cases, but recommends that where it is necessary to wean the baby (this he does not consider necessary in the case

[1] At the Evelina Children's Hospital.

of private patients) it should be done some weeks before the operation, so that the baby may be well accustomed to artificial nourishment by that time. He operates by tying the sac as high as possible, after division of the external oblique, but he has given up as unnecessary the suture of the internal oblique or conjoint tendon to Poupart's ligament. He examined a large number of cases six months to two years after operation, and found no recurrence, but no consecutive statistics of the cases operated on are given. His method of treatment after the operation is interesting. He says "they were placed on a mattress so divided that its middle third was replaced by a bed-pan covered by a rubber air-cushion, which in small babies prevents soiling of the back, and the possibility of excoriation of the skin. The penis was secured into the neck of a test-tube leading to a bottle, so that the urine is carried well away from the region of the wound."

Mr. Clogg[1] is another strong advocate of operation for hernia in children, and reports 126 cases with no mortality. He operates after the fourth month, as soon as the baby can be weaned in hospital practice, but without weaning the infant in private practice. The operation which he performs is practically Bassini's, but, like Bull and Coley, he does not always transplant the cord. He says an attempt had been made to ascertain the after-results of all cases, and he found it possible to do this in 75 per cent. The result, he tells us, was perfect in every case; but he does not give the period which had elapsed since the operation in these cases. All he says is, "The most recent case being operated on three months ago." But we want to know the period since operation in all the cases in considering the question of non-recurrence.

**Reduction en masse of Hernia.**—Mr. Edred Corner and Mr. Howitt[2] find, from an investigation of the record of the cases at St. Thomas's and St. Bartholomew's Hospitals, how rare the occurrence of reduction *en masse* of a strangulated hernia is—only once in every 331 cases. They found no record of reduction *en masse* of an umbilical or ventral hernia. In 4 per cent. the reduction was, curiously enough, spontaneous; in most of the cases from taxis used by the doctor; but in a fair proportion of cases by the patient himself (28 per cent.). The hernia was almost invariably of small intestine. The records of four cases which occurred at St. Thomas's Hospital since 1900 are given, and one is a remarkable case, inasmuch as the reduction of a hernia (a partial enterocele) at operation was incomplete; for at a second operation, required a few days later by persistence of symptoms, the gut was found "partially" strangulated by the

1 *Practitioner*, 1907, lxxix, 364. 2 *Ann. Surg.*, 1908, xlvii, 573.

neck of the sac. It had been recognised that the neck had been torn at the first operation, but not that the bowel was still constricted by it.

The authors consider that reduction *en masse* also occurs in non-strangulated cases, but their description of the condition does not seem very convincing ; some of the examples would seem to be rather the reduction of portions of bowel or bladder uncovered by peritoneum. In one case a piece of omentum seemed to have inverted an adherent portion of the sac, but this is perhaps hardly a reduction *en masse.*

C. A. MORTON.

